# 

**Published:** 1794

**Authors:** 


### MEDICAL FACTS

AND

### OBSERVATIONS.

S.G.O.L. [illegible]

VOLUME THE FIFTH. + VI.

Surgeon Genl's Office
6885
LIBRARY.
Washington D.C.

LONDON:

PRINTED FOR J. JOHNSON, N° 72, ST. PAUL'S CHURCH YARD.

M.DCC.XCIV.

1794

*Catalogue of Books.* — — — 190

*Index.* — — — — — 223

---

DIRECTIONS TO THE BINDER.

Plate the Firſt may be placed at page 95, where the references to it are explained; and Plate the Second at page 98.

---

ERRATUM.

In Vol. IV. page 122, line 1, *for* but the uterus, *read* but the diſcharge from the uterus.

ſeveral medical men, and they ſaw about a ſpoonful of an imperfectly digeſted chylous ſubſtance iſſue from the fiſtulous opening; after this he ſwallowed a glaſs of wine, which was immediately diſcharged through the ſame paſſage. It was obſerved that in other reſpects the patient had the appearance of a man in perfect health.

---

### CATALOGUE OF BOOKS.

1. A Treatiſe on the Dropſy of the Brain, illuſtrated by a Variety of Caſes: to which are added Obſervations on the Uſe and Effects of the Digitalis purpurea in Dropſies. By *Charles William Quin*, M. D. Fellow of the King's and Queen's College of Phyſicians, Phyſician General of his Majeſty's Army in Ireland, and of the Royal Hoſpital for Invalids near Dublin. 8vo. *Murray*, London, 1790.

2. A Practical Diſſertation on the Medicinal Effects of the Bath Waters. By *William Fal-*

*coner*,

*coner*, M. D. F. R. S. and Phyſician to the Bath Hoſpital. 8vo. *Robinſons*, London, 1791.

3. Inſtitutes of Natural Hiſtory; containing the Heads of the Lectures in Natural Hiſtory, delivered by Dr. *Walker*, in the Univerſity of Edinburgh. 8vo. Edinburgh, 1792.

4. A ſketch of a Plan to exterminate the caſual Small Pox from Great Britain, and to introduce general Inoculation; to which is added a Correſpondence, on the Nature of variolous Contagion, with Mr. Dawſon, Dr. Aikin, Profeſſor Irvine, Dr. Percival, Profeſſor Wall, Profeſſor Waterhouſe, Mr. Henry, Dr. Clark, Dr. Odier, Dr. James Currie: and on the beſt means of preventing the Small Pox, and promoting Inoculation, at Geneva; with the Magiſtrates of the Republic. By *John Haygarth*, M. B. F. R. S. Lond. F. R. S. and R. M. S. Edinburgh, and of the American Academy of Arts and Sciences. 2 vols. 8vo. *Johnſon*, London, 1792.

5. An Experimental Inquiry into the conſtituent Principles of the Sulphureous Water at Nottington, near Weymouth: together with Obſervations relative to its application in the Cure of Diſeaſes. By *Robert Graves*, M. D. 8vo. *Johnſon*, London, 1792.

6. Obſervations on the Bark of a particular

Species

Species * of Willow, ſhowing its ſuperiority to the Peruvian, and its ſingular Efficacy in the Cure of Agues, Intermittent Fevers, Hæmorhages, etc. illuſtrated by Caſes. By *Samuel James*, ſurgeon. 8vo. *Johnſon*, London, 1792.

7. Obſervations on Cold Bathing. By *William Simpſon*, ſurgeon at Knareſborough. 8vo. *Johnſon*, London, 1792.

8. Eſſay on the Materia Medica; in which Dr. Cullen's Theories are conſidered. By *James Moore*, ſurgeon. 8vo. *Cadell*, London, 1792.

9. Obſervations on the Blindneſs occaſioned by Cataracts; ſhowing the Practicability and Superiority of a Mode of Cure without an Operation. By *Henry Barry Peacock*. 8vo. *Pridden*, London, 1792.

10. A Guide for Gentlemen ſtudying Medicine at the Univerſity of Edinburgh. By *J. Johnſon* †, Eſq. 8vo. *Robinſons*, London, 1792.

11. An Eſſay on Generation. By *J. F. Blumenbach*, M. D. Aulic Counſellor to his Britannic Majeſty, and Profeſſor of Phyſic in the Univerſity of Gottingen; tranſlated from the German ‡. 12mo. *Cadell*, London, 1792.

* *Salix Latifolia* Linn.

† Fictitious.

‡ By *Alexander Crichton*, M. D.

12. Obſer-

12. Obſervations on the Scurvy; with a Review of the Opinions lately advanced on that Diſeaſe, and a new Theory defended, on the approved Method of Cure, and the Induction of Pneumatic Chemiſtry; being an Attempt to inveſtigate that Principle in recent vegetable Matter, which, alone, has been found effectual in the Treatment of this ſingular Diſeaſe; and from thence to deduce more certain Means of Prevention than have been adopted hitherto. Second Edition. By *Thomas Trotter*, M. D. Surgeon of his Majeſty's Ship the Duke. 8vo. *Longman*, London, 1792.

13. Medical Botany; containing Syſtematic and General Deſcriptions, with Plates, of all the Medicinal Plants, indigenous and exotic, comprehended in the Catalogues of the Materia Medica, as publiſhed by the Royal Colleges of Phyſicians of London and Edinburgh, accompanied with a circumſtantial Detail of their medicinal Effects, and of the Diſeaſes in which they have been moſt ſucceſsfully employed. By *William Woodville*, M. D. of the Royal College of Phyſicians, London. In 3 vols. 4to. *Phillips*, London, 1790—3.

14. Experiments on Animal Electricity, with their Application to Phyſiology; and ſome Pathological and Medical Obſervations. By *Euſe-*

*bius Valli*, M. D. Correſponding Member of the Royal Academy of Sciences of Turin. 8vo. *Johnſon*, London, 1793.

15. Experiments and Obſervations relative to the Influence lately diſcovered by M. Galvani, and commonly called Animal Electricity. By *Richard Fowler*. 8vo. Edinburgh, 1793.

16. An Account of the Veterinary College, from its Inſtitution in 1791. 8vo. *Phillips*, London, 1793.

17. A Treatiſe on the Structure, Economy, and Diſeaſes of the Liver; together with an Inquiry into the Properties and component Parts of the Bile and biliary Concretions; being the Subſtance of the Gulſtonian Lectures read at the College of Phyſicians, in the Year 1792. By *William Saunders*, M. D. Fellow of the College of Phyſicians, of the Royal Societies of London and Edinburgh, and Senior Phyſician to Guy's Hoſpital. 8vo. *Robinſons*, London, 1793.

18. Experiments and Obſervations on the Anguſtura Bark. By *Auguſtus Everard Brande*, Apothecary to the Queen. The ſecond Edition enlarged. 8vo. *Payne*, London, 1793.

19. Of the Hot-Waters near Briſtol. By *John Nott*, M. D. 8vo. Briſtol, 1793.

20. Obſervations on the Nature and Cure of Calculus, Sea-Scurvy, Conſumption, Catarrh, and and Fever; together with Conjectures upon several other Subjects of Physiology and Pathology. By *Thomas Beddoes*, M. D. 8vo. *Murray*, London, 1793.

21. A letter to *Erasmus Darwin*, M. D. on a new Method of treating Pulmonary Consumption, and some other Diseases hitherto found incurable. By *Thomas Beddoes*, M. D. 8vo. *Murray*, London, 1793.

22. Practice of Physic; wherein is attempted a concise Exposition of the Characters, Symptoms, Causes of Diseases, and Method of Cure; with Formulæ, in which such new Articles are introduced as seem from Trial to be valuable Additions to the Materia Medica: with a copious Index of Diseases and Medicines; the whole intended for the Assistance of the young Practitioner. By *Richard Temple*, M. D. Licentiate of the Royal College of Physicians, London. 8vo. *Johnson*, London, 1792.

23. New Experiments with Mercury on the Small-Pox, by which is demonstrated its specific Virtue in that Disease. By *F. Van Woensel*, M. D. translated from the French, by *William Fowle*, M. D. 8vo. *Crowder*, London, 1793.

24. Observations on the History and Cure of the Asthma; in which the Propriety of using the

Cold Bath in that Diforder is fully confidered. By *Michael Ryan*, M. D. and Member of the Royal Antiquarian Society of Edinburgh. 8vo. *Robinfons*, London, 1793.

25. The Morbid Anatomy of fome of the moft important Parts of the Human Body. By *Matthew Baillie*, M. D. F. R. S. Fellow of the Royal College of Phyficians, and Phyfician of St. George's Hofpital 8vo. *Johnfon*, London, 1793.

26. The Caufes of the great Number of Deaths amongft Adults and Children, in putrid, fcarlet Fevers, and ulcerated fore Throats, explained; with more fuccefsful Modes of treating thofe alarming Diforders, as practifed at the St. Mary-le-Bone Infirmary. By *William Rowley*, M. D. 8vo *Newbery*, London, 1793.

27. An Appendix to a Treatife on the Hydrocele *. By *James Earle*, Efq. Surgeon Extraordinary to his Majefty's Houfehold, and Senior Surgeon to St. Bartholomew's Hofpital. 8vo. *Johnfon*, London, 1793.

28. Practical Obfervations on the Operation for the Stone, in which an Affertion of the late Dr. Auftin is confidered; with a Preface containing fome Account of Dr. Auftin's Life. By *James Earle*, Efq. 8vo. *Johnfon*, London, 1793.

* See Volume II. page 208.

29. A Diſſertation on the Structure of the Obſtetric Forceps, pointing out its Defects, and eſpecially thoſe with the double curved Blades; at the ſame Time, ſhewing particularly the ſafe Application of thoſe with ſingle curved Blades, as geometrically proportioned and conſtructed: and likewiſe, ſhewing the Neceſſity and good Effects of ſeveral new Forms of the ſingle curved Blade, as the narrow, fanged, and refluted, in certain Caſes of retarded Labours; together with Cautions, Remarks, and Reflections on the Conduct and Management of Labours in general. By *R. Rawlins*, Surgeon, Oxford. 8vo. *White*, London, 1793.

30. Practical Eſſays on the Management of Pregnancy and Labour, and on the inflammatory and febrile Diſeaſes of lying-in Women. By *John Clarke*, M. D. Phyſician to the Lying-in Hoſpital in Store-ſtreet, and to the Aſylum for female Orphans; and Teacher of Midwifery in London, 8vo. *Johnſon*, London, 1793.

31. An Eſſay towards a Definition of animal Vitality; read at the Theatre, Guy's Hoſpital, January 26, 1793; in which ſeveral of the Opinions of the celebrated John Hunter are examined and controverted. By *John Thelwall*,

Member of the Phyfical Society, &c. 4to. *Robinfons*, London, 1793.

32. Facts * tending to fhow the Connection of the Stomach with Life, Difeafe, and Recovery. 8vo. *Murray*, London, 1793.

33. Friendly Cautions to the Heads of Families, and others, neceffary to be obferved, in order to preferve Health and Long life. Containing, alfo, ample Directions to Nurfes who attend the Sick, Women in Child-bed, &c. By *Robert Wallace Johnfon*, M. D. at Brentford. 12mo. *Robinfons*, London, 1793.

34. A Pofologic Companion to the London Pharmacopoeia. 12mo. *Johnfon*, London, 1793.

35. Advice to Parents on the Management of their Children in the Small-Pox, and during Inoculation: with a few Cafes confirming the Author's Opinion. To which are added, fome general Obfervations on the Ufe of Tea, and the prefent Regimen of Diet among the higher Ranks of Society. 8vo. *Robinfons*, London, 1793.

36. Lectures on the Elements of Farriery, on the Art of Horfe-fhoeing, and on the Difeafes of the Foot. Defigned chiefly for the ufe of the

* By *Charles Webfter*, M. D.

Pupils

Pupils of the Veterinary College, London. By *Charles Vial de Sainbel*, Profeſſor of Veterinary Medicine in the College. 4to. *Bell*, London, 1793.

37. The Language of Botany; being a Dictionary of the Terms made uſe of in that Science, principally by Linnæus: with familiar Explanations, and an Attempt to eſtabliſh ſignificant Engliſh Terms. The whole interſperſed with Critical Remarks. By *Thomas Martyn*, B. D. F. R. S. Profeſſor of Botany in the Univerſity of Cambridge. 8vo. *White*, London, 1793.

38. Indigenous Botany; or Habitations of Engliſh Plants: containing the Reſult of ſeveral Botanical Excurſions, chiefly in Kent, Middleſex, and the adjacent Counties, in 1790, 1791, and 1792. By *Colin Milne*, L. L. D. Author of a Botanical Dictionary, and Inſtitutes of Botany; and *Alexander Gordon*, Reader on Botany in London. Vol. I. 8vo. *Lowndes*, London, 1793.

39. Popular Obſervations on apparent Death from Drowning, Suffocation, &c. with an Account of the Means to be employed for Recovery. Drawn up at the Deſire of the Northamptonſhire Preſervative Society. By *James Curry*, M. D. Phyſician to the Northampton Hoſpital. 8vo. *Law*, London, 1793.

40. Obſervations on the Effects of Buxton

Waters. By *Joseph Denman*, M. D. 8vo. *Johnson*, London, 1793.

41. A plan for preventing the fatal Effects from the Bite of a Mad-Dog, with Cases. By *Jesse Foot*, Surgeon. 8vo. *Becket*, London, 1793.

42. Observations on the Disease of the Hip-Joint; to which are added, some Remarks on White Swellings of the Knee, the Caries of the Joint of the Wrist, and other similar Complaints; the whole illustrated by Cases, and Engravings taken from the diseased Parts. By *Edward Ford*, F. S. A. Surgeon to the Westminster General Dispensary. 8vo. *Dilly*, London, 1793.

43. Oratio Anniversaria in Theatro Collegii Regalis Medicorum Londinensium, ex Harveii Instituto habita, A. D. 1790, Festo divi Lucæ, a *Joanne Ash*, M. D. Coll. Reg. Med. Lond. Soc. R. et. S. A. Socio. 4to. Londini, 1790.

44. Floræ Cantabrigiensi Supplementum tertium. Auctore *Richardo Relhan*, A. M. Collegii Regalis Capellano, Villæ de Hemingby in Agro Lincolniensi Rectore, Regiæ Societatis Londinensis et Societatis Linneanæ Socio. 8vo. Cantabrigiæ, 1793.

45. Dis-

45. Differtatio Inauguralis de Afcite. Auctore *Joanne Barry*, Hiberno. 8vo. Edinburgi, 1792.

46. Differtatio Inauguralis de Gonorrhœa virulenta. Auctore *Georgio J. Binham*, Jamaicenfi. 8vo. Edin. 1792.

47. Differtatio Inauguralis de Apoplexia. Auctore *Joanne Bolton*, A. B. Hiberno. 8vo. Edin. 1792.

48. Differtatio Inauguralis de Scrophula. Auctore *Gulielmo Bullen*, Hiberno. 8vo. Edin. 1792.

49. Differtatio Inauguralis de Vita Submerforum refufcitanda. Auctore *Jacobo Davidfon*, Scoto. 8vo. Edin. 1792.

50. Differtatio Inauguralis de Febre flava Indorum Occidentalium. Auctore *Joanne Eften*, Bermudenfi. 8vo. Edin. 1792.

51. Differtatio Inauguralis de Scorbuto. Auctore *Georgio Goodman*, Anglo. 8vo. Edin. 1792.

52. Differtatio Inauguralis de Dyfpepfia. Auctore *Gulielmo Ledger*, Hiberno. 8vo. Edin. 1792.

53. Differtatio Inauguralis de Dyfenteria. Auctore *Gulielmo B. Lewis*, Virginienfe. 8vo. Edin. 1792.

54. Dif-

54. Differtatio Inauguralis de Rheumatifmo acuto. Auctore *Jacobo Drew M'Caw*, Virginienfe. 8vo. Edin. 1792.

55. Differtatio Inauguralis de Afcite Abdominali. Auctore *Jofepho Miller*, Hiberno. 8vo. Edin. 1792.

56. Differtatio Inauguralis de Apoplexia. Auctore *Philippo Syng Phyfick*, Philadelphienfi. 8vo. Edin. 1792.

57. Differtatio Inauguralis de Dyfenteria. Auctore *Joanne Stennet*, Jamaicenfi. 8vo. Edin. 1792.

58. Differtatio Inauguralis de Ictero. Auctore *Gulielmo Vavafour*, Anglo. 8vo. Edin. 1792.

59. Differtatio Inauguralis de Amenorrhœa. Auctore *Joanne Weems*, Marylandienfi. 8vo. Edin. 1792.

60. Differtatio Inauguralis de Dyfpepfia. Auctore *Alexandr. Philip. Wilfon*, Scoto. 8vo. Edin. 1792.

61. Differtatio Inauguralis de Variolis. Auctore *Samuele Wilfon*, Virginienfe. 8vo. Edin. 1792.

62. Bibliothecæ Collegii Regii Medicorum Edinburgenfis Catalogus, fecundum Auctorum Nomina difpofitus. 8vo. Edin. 1793.

63. *Jo-*

63. *Joannis Andreæ Murray*, Equitis Ord. Suecic. de Wasa, Prof. Gottingens. &c. Enumeratio Librorum præcipuorum medici Argumenti; recudi curavit et permulta Additamenta adjecit *Frid. Guil. vom Halem.* 8vo. Aurich. 1792.

64. *Joannis Andreæ Murray*, Equitis Ord. R. de Wasa, Profess. Medic. et Botan. ord. in Acad. R. Gotting. &c. Apparatus Medicaminum tam simplicium quam præparatorum et compositorum in Praxeos Adjumentum consideratus. Volumen sextum, post Mortem Auctoris edidit *Lud. Christoph. Althof*, M. D. 8vo. Gottingæ, 1792.

65. Dissertatio Inauguralis de Cortice Caribæo Cortici peruviano substituendo. Auctore *Fried. Wilhelm. Aufmkolk.* 8vo. Gottingæ, 1792.

66. Dissertatio Medica Inauguralis qua inquiritur in Theoriam Weikhardianam de Hydrope Cerebri, nec non in veram ejusdem Morbi Indolem. Auctore *Joh Georg. Lodemann.* 8vo. Gottingæ, 1792.

67. Dissertatio Inauguralis sistens veram in Medicamentorum Vires inquirendi Rationem. Auctore *Joh. Dan. Droop.* 8vo. Gotting. 1792.

68. Dissertatio Inauguralis de Asphyxia Neo-

 natorum.

natorum. Auctore *Johann. Ulrich von Waldkirch.* 8vo. Gottingæ, 1792.

69. Dissertatio Inauguralis de Auditu difficili. Auctore *Joh. Fried. Kritter*, Gottingensi. 8vo. Gottingæ, 1792.

70. Dissertatio Inauguralis sistens Infarctus Differentias. Auctore *Christ. Jac. Friedr. Rüst.* 8vo. Gottingæ, 1792.

71 Observationes de Virtute Corticis Geoffrææ Surinamensis contra Tæniam. Auctore *Augusto Jacobo Schwartze*, Hanoviense. 4to. Gottingæ, 1792.

72. *Georg. Christ. Bonhard*, Hassia-Darmstadiensis, Diss. Inaug. de usu Lienis verisimillimo. 4to. Jena, 1792.

73. Dissert. med. Inaug. de Suicidio in Foro medico non semper culposo. Auctore *Joh. Eberhard Gagel.* 4to. Jena, 1792.

74. Dissertatio Inauguralis medica de Rebus Pathematum in Specie Gaudii in Corpus humanum Efficaciam moderantibus. Auctore *J. G. Kleefeld*, Gedanensi. 4to. Jena, 1792.

75. Dissertatio Inauguralis de usu Glandulæ Thymi verisimillimo. Auctore *Gottl. Karch.* 4to. Jena, 1792.

76. Dissertatio Inauguralis de Operationibus atque Viis Medicamentorum externorum Stases

Syftematis lymphatici fubmoventium. Auctore *Johanne Georgio John*. 4to. Jena, 1792.

77. Differtatio Inauguralis de Hydrometra. Auctore *Johanne Petro Kommer*. 4to. Jena, 1792.

78. Differtatio Inauguralis de Annis Climactericis. Auctore *Philippo Daniele Benjam. Seifert*. 4to. Jena, 1792.

79. Differt. Inaug. Anatomico-Phyfiologica qua demonftratur Cor Nervis carere: addita Difquifitione de Vi Nervorum Arterias cingentium. Auctore *J. B. J. Behrends*, Moeno-Francofurtano. 4to. Moguntiæ, 1792. c. tabula æn.

80. Differtatio Inauguralis medica de Fracturis Offium Pelvis. Auctore *C. C. Creve*, Confluo. 4to. Moguntiæ 1792.

81. Differtationes medicæ in Univerfitate Vindobonenfi habitæ ad Morbos chronicos pertinentes et ex *Max. Stollii* Prælectionibus potiffimum confcriptæ. Edidit et præfatus eft *Jofephus Eyerel*. Volumen quartum et ultimum. 8vo. Viennæ, 1792.

82. *Jofephi Eyerel* Commentaria in *Maximiliani Stollii* Aphorifmos de cognofcendis et curandis Febribus. Tomus quintus. 8vo. Viennæ, 1792.

83. Apparatus Medicaminum ad ufum Nofocomii Ticinenfis. 8vo. Pavia, 1790.

84. *Baf-*

84. *Bassiani Carminati*, Hygiene Therap. gener, Mat. Med. et Chirurg. ac Pharmaciæ in Ticin. Gymnas. reg. Professoris, Nosocomii Med. primar. &c. Hygiene Therapeutice, et Materia Medica. Vol. I. 8vo. Pavia, 1791.

85. *Joh. Diet. Eggert* Commentatio de Virtute anthelmintica Geoffrææ Surinamensis adjectis Observationibus recentioribus. 8vo. Marburg, 1791

86. *Anton.* L. B. de *Störck* Præcepta medico-practica in usum Chirurgorum castrensium et ruralium Ditionum Austriacarum, e Lingua Germanica in Latinam versa per *J. M. Schosulan*, Facultatis medicæ Vindobonensis p. t. Decanum. Editio altera aucta. Tom. II. 8vo. Viennæ, 1791.

87. Critices Semiologiæ medicinalis Rudimenta pro Gradu Doctoris exhibet *Didericus Fridericus Buettner*, Medicinæ Practicus apud Palæo-Stretlitienses. 8vo. Rostochii, 1791.

88. Braschiæ Plantæ novi Generis Descriptio, Nomini Majestatique Pii sexti P. M. dicata; Auctore *Nicol. Martelli*, Med. Doct. et Botan. Prof. 4to. Romæ, 1791.

89. Scriptores Neurologici, sive Opera minora ad Anatomiam, Physiologiam, et Pathologiam

Ner-

Nervorum ſpectantia. Tom. II. 4to. Lipſiæ, 1792. c. tabulis æneis.

90. Theſaurus Semiotices pathologicæ, quem collegit atque edidit *Joannes Chriſt. Traug. Schlegel*, Sereniſſimi Principis de Schönburg Conſil. Aulic. et Archiater. Vol. II. 8vo. Stendal, 1792.

91. *Chriſt. Georg. Ludov Gottlieb Duiſburg*, Schauenburgo-Lippiac. Diſſertatio Inauguralis ſiſtens Phyſiologiam et Pathologiam Uteri. 4to. Jena, 1792.

92. Epiſtola gratulatoria ad Viros illuſtres et experientiſſimos Dominos Doctores et S. R. I. liberæ Reip. Norimbergenſis Phyſicos ordinarios, nec non inclyti Collegii medici hac in Urbe CC. Annos feliciter florentis, hodieque Sæcularia ſacra ſolenni Actu celebrantis, Aſſeſſores ſpectatiſſimos, qua animum ſuum hoc feſto Die exultantem, devota Mente, ſummoque Obſervantiæ Cultu, teſtari, ſimulque Narrationem ſuccinctam de Vita et Meritis *Joach. Camerarii*, Conditoris hujuſce celeberrimi Collegii adnectere, conatur Auctor *Paulus Sigiſmundus Carolus Preu*, Med. Stud. 4to. Altdorfii, 1792.

93. *Johann. Kämpf*, M. D. Enchiridium medicum paſſim emendatum et auctum; denuo edidit edidit D. *C. Th. Kortum*, Med. Stollberga-Juliacensis. 8vo. Francofurti ad Mœnum, 1792.

94. Pharmacopœia in Usum Officinarum Reipublicæ Bremensis conscripta. 8vo. Bremen. 1792.

95. *Francisci de Paula Schrank*, Theologiæ et Philosophiæ Doctoris, Serenissimo ac potentissimo Electori Palatino-Bavaro a Consiliis Ecclesiasticis, in Universitate Ingolstadiensi Professoris, Primitiæ Floræ Salisburgensis; cum Dissertatione prævia de Discrimine Plantarum ab Animalibus. 8vo. Francofurti ad Moenum, 1792.

96. Dispensatorium Lippiacum Genio moderno accomodatum; Auctoritate Collegii medici redegit *Joannes Christianus Friedericus Scherf*, M. et Ch. D. S. Princip. Lipp. Aulæ Consil. et Medicus, Collegii Med. Sodalis, &c. Pars prima. 8vo. Lemgo, 1792.

97. Historia Epidemiæ variololæ Erlangensis Anni 1790; Auctore D. *Joann. Maximil. Plinta.* 8vo. Erlangen, 1792.

98. Exercitationes Academicæ Argumenti aut anatomici aut physiologici, quas ex Dissertationum Regiomontarum Penu in Fasciculum collegit *Joh. Dan. Metzger*. 8vo. Regiomonti, 1792.

99. *J.*

99. *Joannis Christiani Reil*, Med. et Chirurg. D. Prof. Therap. p. o. Directoris Scholæ clinicæ, Civit. Halens. Physic. Memorabilium Clinicorum medico-practicorum, Vol. II. Fascic. II. 8vo. Halæ, 1792.

100. *Seb. Joann. Lud. Dovering*, Commentatio medica de Vomitu. 8vo. Marburg, 1792.

101. De Electrici Ignis Natura Dissertatio ab *Josepho Gardinio*, Philosophiæ et Med. Doct. Domo Alba Pompeia reg. Scient. et Literar. Acad. Mantuanæ exhibita 1788 ab eademque probata. 4to. Mantua, 1792.

102. Dissertatio Physiologica Inauguralis de Structura et Usu Vasorum Absorbentium. Auctore *Edvardo Holme*, Anglo: e Soc. Med. Edin. Soc. Litt. et Philos. Mancuniens. Litterarum Commercio Conjuncto. 4to. Lugduni Batavorum, 1793.

103. Dissertatio Inauguralis de Signis Morborum quæ ex Oculorum Habitu petuntur. Auctore *Lebr. Christ. Fabricio*, Magdeburg. 8vo. Halæ, 1793.

104. Dissertatio Inauguralis de Irritabilitatis Notione, Natura, et Morbis. Auctore *Lud. Gautier*, Vratislav. 8vo. Halæ, 1793.

105. De Baryte muriato Specimen secundum, quo medica hujus Salis Historia proponi-

 tur.

tur. Auctore *Joh. Aug. Schmidt*, Med. Baccal. 8vo. Lipsiæ, 1793.

106. Dissertatio Inauguralis de Febre Scarlatina. Auctore *Eustachio a Khittel.* 8vo. Halæ, 1793.

107. Dissertatio Inauguralis de Febris nervosæ lentæ Pathologia. Auctore *Paul. Sigism. Brosig.* 8vo. Halæ, 1793.

108. Dissertatio Inauguralis Meletamata critica in Theoriam illustris Reillii de Morbis Venereis sistens. Auctore *Joh. Aug. Chemnitz.* 8vo. Halæ, 1793.

109. Dissertatio Inauguralis de Prosopalgia. Auctore *Salomon. Simon.* 8vo. Halæ, 1793.

110. Dissertatio Inauguralis qua Quæstiones quædam de Suppurationis Indole ventilantur. Auctore *Joh. Dan. Arnold.* 8vo. Halæ, 1793.

111. Dissertatio Medica de Cortice Angusturæ quam Præside *Carol. Petr. Thunberg*, Eq. Aur. Reg. Ord. de Wasa, Med. Doct. Prof. Med. et Botan. &c. pro Gradu Doctoris publice ventilandam sistit *Carolus Fr. Blumenberg*, Stip. Nessel. Dalekarlus. 4to. Upsaliæ, 1793.

112. Projet de Reforme sur l'Exercise de la Medecine en France; par M *Ant. Petit*, Docteur Regent de la Faculté de Medecine de Paris, de l'Academie des Sciences, ancien Professeur d'Anad'Anatomie et de Chirurgie au Jardin du Roi. 8vo. Paris, 1790.

113. Phytozoologie philoſophique, dans laquelle on demontre comment le Nombre des Genres et des Eſpeces, concernant les Animaux et les Vegetaux, a été limité et fixé par la Nature; avec les Moyens de donner l'Hiſtoire la plus complette et la plus parfaite de ces Corps organiſés differens ſelon la Decouverte du Syſteme Naturel: par *Noel Joſeph de Necker*, Botaniſte de S. A. S. E. Bavaro-palatine; Hiſtoriographe du Palatinat du Rhin et des Duchés de Berg et Juliers; Membre de l'Academie des Sciences de Manheim, et Aſſocié de diverſes Academies des Sciences de l'Europe. 8vo. Neuwied ſur le Rhin, 1790.

114. L'Homme Phyſique et Moral, ou Recherches ſur les Moyens de rendre l'Homme plus ſage, et de le garantir des diverſes Maladies qui l'affligent dans ſes differens âges; par M. *Ambr. Ganne*, Doćteur en Philoſophie, ancien Chirurgien-aide-Major des Hopitaux de la Marine et des Caribiniers. 8vo. Straſbourg, 1791.

115. Traité de la Gonorrhée, et des Maladies des Voies Urinaires qui en ſont la ſuite; dans lequel on indique des nouvelles Bougies medicamenteufes pour les guerir. Par *Fr. Teytaud*, Chirurgien a Paris, et Chirurgien-Major de la Garde Nationale. 12mo. Paris, 1791.

116. Cours Complet ou Traité des Fievres. Par M. *Grimaud*, Profeffeur dans l'Univerfité de Montpellier. Tom. I. 8vo. Montpellier, 1791.

117. Memoire de Medecine pratique fur les Efforts, comme Principes de plufieurs Maladies, tant aigües que chroniques. Par M. *Balme*, Medecin au Puy. 12mo. Paris, 1791.

118. Hiftoire des Champignons de la France, ou Traité elementaire, renfermant dans un Ordre methodique les Defcriptions et les Figures des Champignons qui croiffent naturellement en France. Par M. *Bulliard*. Tome Premier, Fol. Paris, 1791.

119. Obfervations de Phyfique et de Medecine, faites en differens Lieux de l'Efpagne ; on y a joint des Confiderations fur la Lepre, la petite Verole, et la Maladie venerienne. Par M. *Thiery*, Docteur-Regent de la Faculté de Medecine de Paris, Medecin Confultant du Roi, 2 tomes. 8vo. Paris, 1792.

120. Actes de la Société d'Hiftoire Naturelle de Paris. Tome premier. Premiere Partie. Folio. Paris, 1792. With 13 Plates.

121. Me-

121. Memoires sur les grandes Gelées et leurs Effets; où l'on essaie de determiner ce qu'il faut croire de leurs Retours periodiques, et de la Gradation en plus ou moins du Froid de notre Globe. Par M. l'Abbé *Mann*, Chanoine de l'Eglise de N. D. a Courtray; Membre de la Commission Royale des Etudes: Membre et Secretaire perpetuel de l'Academie Imperiale et Royale des Sciences et Belles Lettres de Bruxelles; Membre de la Société Royale de Londres, de l'Academie Electorale Palatine de Manheim, des Sociétés de Milan, Liege, Rotterdam, Zelande, &c. 8vo. Gand, 1792.

122. Verhandelingen uitgegeeven door de Maatschappy ter Bevordering van den Landbouw te Amsterdam. Negende Deel, *i. e.* Essays published by the Society for the Improvement of Agriculture at Amsterdam. Volume the ninth *. 8vo. Amsterdam, 1793.

* The volume here announced, consists of a single Essay, the author of which received the gold medal offered by the Society, in 1788, for the best treatise on the subject. It is entitled "Verhandeling over het Verlossen der Koei-"jen; door *Johan Gunther Eberhard*, Med. Doctor en "Vroedmeester te Zeist; met Plaaten." *i. e.* "A Treatise "on the Parturition of Cows; by *John Gunther Eberhard*, "M. D. and Man Midwife at Zeist; with 12 Plates."

 123. Bey-

123. Beytrag zur Geschichte epidemischer Gallen Fieber. *i. e.* Materials for a History of an Epidemic Bilious Fever. By *Charles Christ. Eckner*, M. D. Physician to the Garrison at Schwarzbourg-Rudolstadt, and Member of the Imperial Academy of Nat. Curios. 4to. Leipsic, 1790.

124. Versuch einer Beschreibung der hauptsæchlichen in Reval herschenden krankheiten. *i. e.* Essay towards a Description of the principal Diseases that prevail at Reval. By *Hermann Bluhm*, M. D. 8vo. Marbourg, 1790.

125. Ueber die Vortheile die dem Staat aus einer Schule der Gerichtlichen Arzneywissenschafft zuflieſſen. *i. e.* On the Advantages which a State derives from a School of Medical Jurisprudence. By *F. G. Wezeler*, Doctor of Physic and Surgery, Professor of Medical Jurisprudence and of Midwifery in the University of Bonn. 8vo. Bonn, 1790.

126. Von den Vortheilen der Krankenhausen für den Staat. *i. e.* Of the Advantages of Hospitals to the State. By *Adalbert Frederick Marcus*, first Physician of the General Hospital at Bamberg. 8vo. Bamberg, 1790.

127. Ueber die Cretinen, eine besondere Menschenabart in den Alpen. *i. e.* On the

Cretins, a particular degenerate Race of Men in the Alps. By *J. F. Ackermann.* 8vo. Gotha, 1790, with Copper Plates.

128. Verſuch die Metamorphoſe der Pflanzen zu erklären. *i. e.* An Attempt to explain the Metamorphoſes of Plants. By *J. W. von Goethe,* Privy Counſellor to the Duke of Saxe-Weimar. 8vo. Gotha, 1790.

129. Chemiſche Unterſuchung der Mineralquellen zu Carlſbad. *i. e.* Chemical Examination of the Mineral Springs at Carlſbad. By *Martin Henry Klaproth.* 8vo. Berlin, 1790.

130. Beyträgen zur gerichtlichen Arzneygelahrheit und zur mediciniſchen Polizey. *i. e.* Collections relative to medical Juriſprudence and Police. By *William Henry Sebaſtian Bucholz,* Counſellor of the Mines, and Phyſician to the Duke of Saxe-Weimar, 3 vol. 8vo. Weimar, 1782-90

131. Abhandlung von der Umbeugung der Gebährmutter. *i. e.* A Treatiſe on the retroverted Uterus. By *John Melitch,* M. D. and Practitioner of Midwifery at Prague. 8vo. Prague, 1790.

132. Lehrſätze des Chirurgiſchen Verbandes. *i. e.* Principles of Surgical Bandages. By *Francis Joſeph Hofers,* public Teacher of Anatomy.

tomy. 8vo. Erlangen, 1790. Part. I. with six Copper Plates.

133. Neues Archiv der Praktischen Arzneykunst, fur Aerzte, Wundärzte, und Apotheker, von verschiedern Verfassern. *i. e.* New Archives of the Practice of Physic, for Physicians, Surgeons, and Apothecaries, from different Authors. By Professor *Meckel*, of Halle. 8vo. Leipsic, 1790.

134. Archiv fur die Geschichte der Arzneykunde in ihrem ganzen Umfange. *i. e.* Archives for the History of Physic in all its Branches. By *P. L. Wittwer*, M. D. Vol. I. Part I. 8vo. Nurnberg, 1790.

135. Ueber die Durchkreuzung der Sehenerven. *i. e.* On the Decussation of the Optic Nerves. By *Philip Michaelis*; with some Observations by *S. T. Soemmering*, M. D. &c. 8vo. Halle, 1790.

136. Medicinische Skizzen. *i. e.* Medical Sketches. By *J. C. K. Ackermann*, Physician at Zeiz. Part I. 8vo. Leipsic, 1790.

137. Materia Medica; oder Abhandlung von den auserlesenen Arzneymitteln, nebst derselben Ursprung, Gute, Bestandtheile, Masse und Art zu wirken, nebst Vorschriften, wie dieselben aus der Apotheke zu verordnen. *i. e.*

Materia

Materia Medica; or a Treatiſe on the moſt approved Remedies, their Origin, Virtues, conſtituent Parts, Doſe and Mode of operating, together with Preſcriptions, how to order them from the Apothecary. By *John Lewis Lebe-recht Loeſeke*, M. D. The ſixth Edition, corrected and enriched with the lateſt Diſcoveries, by *John Frederick Gmelin*, M. D. 8vo. Berlin, 1790.

138. Tabellariſche Verzeichniſs der einfachen Arzneymittel des Gewächs Reiches; nebſt jedes Gewächs officineller ſowohl als ſyſtematiſcher Benennung, Vaterlande, Sammlungſzeit, Eigenſchaften, Beſtandtheilen, arzneylichen Kräften, Anwendung, &c. in alphabetiſcher Ordnung. *i. e.* Tabular Catalogue of ſimple Remedies of the vegetable Kingdom; with the officinal and ſyſtematic Name of each Plant, its Place of Growth, Seaſon for gathering it, its Qualities, conſtituent Parts, medicinal Virtues, Uſe, &c. the whole arranged in alphabetical Order. By *William Geſenius*, M. D. Phyſician at Nordhauſen. Folio. Stendal, 1790.

139. Abhandlung uber die Wirkungen der Starkſten und reinſten Pflanzenſäure als auſſerliches Heilmittel. *i. e.* Treatiſe on the Effects of of the ſtrongeſt and pureſt vegetable Acid as an external Remedy. 8vo. Leipſic, 1791.

140. Beobachtungen ueber die Krätze geſammlet in dem Arbeitſhauſe zu Prag. *i. e.* Obſervations on the Itch, collected in the Workhouſe at Prague. By *E. F. Guldener von Lobes*, M D. 8vo. Prague, 1791.

141. Von der Caſtration. *i. e.* Of Caſtration. By *Francis L. Marſchal*, Surgeon at Salzburg. 8vo. Salzburg, 1791.

142. Von der Geduld, beſonders des Arztes am Krankenbette; ein Aufsatz, veranlaſſt durch die funfzigjährige Jubelfeyer eines verdienten Arztes. *i. e.* Of Patience, particularly that of the Phyſician at the Bedſide of the Sick; an Eſſay occaſioned by the Celebration of a Fifty-Years Jubilee of a deſerving Phyſician. 8vo. Frankfort on the Mayne, 1791.

143. Vermiſchte mediciniſch-chirurgiſche Schriften. *i. e.* Miſcellaneous medico-chirurgical Writings. By *J. F. Böttcher*. 8vo. Konigſberg, 1791.

144. Verſuch einer allgemeinen Geſchichte des Keichhuſtens. *i. e.* An Eſſay towards a general Hiſtory of the Chin-cough. By *F. G. Danz*, Demonſtrator of Anatomy at Gieſſen. 8vo. Marburg, 1791.

145. Lehrſätze der medicinischer Polizeywiſſenſchaft. *i.e.* Principles of Medical Police. By *Ern. Benj. Gottlieb Hebenſtreit*, M. D. Profeſſor of Phyſic at Leipſic. 8vo. Leipſic, 1791.

146. Annalen des Kliniſchen Inſtituts zu Berlin. *i.e.* Annals of the Clinical Inſtitution at Berlin. By *J. F. Frize*, M. D. 8vo. Berlin, 1791.

147. Handbuch ueber die Veneriſchen Krankheit. *i.e.* A Manual on the Venereal Diſeaſe. By *J. F. Frize*, M. D. 8vo. Berlin, 1791.

148. Neue Chemiſche Nomenklatur für die Deutſche Sprache. *i.e.* A New Chemical Nomenclature for the German Language. By *Chriſtopher Girtanner*, M. D. &c. 8vo. Berlin, 1791.

149. Onomatologia Chymico-practica; oder vollſtandig praktiſcher Handbuch der Chemie in alphabetiſcher Ordnung. *i.e.* A complete practical Manual of Chemiſtry in alphabetical Order. By *H. W. Rels*: with a Preface by *J. Fr. Gmelin.* 8vo. Ulm, 1791.

150. Beſchreibung des Fachinger Mineral-Waſſers, und ſeiner heilſamen Wirkungen. *i. e.* A Deſcription of the Mineral Water at Fachinger, chinger, and its ſalutary Effects, By *Maurice Gerhard Thilenius*, M. D. 8vo. Marburg, 1791.

151. Vom Baue des Menſchlichen Körpers. *i. e.* Of the Structure of the Human Body. By *S. T. Soemmering*, M. D. et Prof. &c. 1ſt, 2d, 3d, and 5th Parts. 8vo. Franckfort on the Mayne, 1791.

152. Ueber der Gebrauch und die Kräfte der Sauren Schwererde in verſchieden Krankheiten. *i. e:* On the Uſe and Effects of the Muriated Barytes in different Diſeaſes. By *C. Wilh. Hufeland*, M. D. Phyſician to the Duke of Saxe-Weimar. 4to. Erfurt, 1792.

153. Ueber die Natur und den Gebrauch der Bäder: *i. e.* On the Nature and Uſe of Baths, By *Henry Matthias Marcard*, Body Phyſician to the Duke of Holſtein-Oldenburg, &c. 8vo. Hanover, 1793.

154. Della ſalutare Iſpirazione Ragionamento filoſofico Critico ſulla Trasfuſione degli Aliti umani per ſoccorſo della Salute. 8vo. Nizza, 1788.

155. Dei Segni della Verginita preſſo gli Antichi. Lettera di D. *G. A.* al Sig. *A. F.* 8vo. Montalbano, 1790.

156. Ammaeſtramenti intorno ai Parti pubblicati d'ordine de Nobili Provveditori alla

Sanita

Sanita di Bergamo a Inſtruzione delle Comaſi principalmente di Campagna. 8vo. Bergamo, 1790.

157. Piano della Scuola Clinica oſſia Inſtruzione per gli Scolari Clinici del Profeſſore Clinico *Nicolo Olivari*, approbata e ſtabilita in Genova dagli Eccellentiſſimi ed Illuſtriſſimi Signori dodeci Protettori dello Spedale grande l'Anno 1789. Seigne il Diſcorſo dell' inſigne Profeſſore Clinico *Cullen*, ſulla Maniera di ſtudiere la Medicina pratica. 8vo. Genoa, 1790.

158. Saggio di alcune Ricerche ſu i Principi e ſulle Virtu della Radice di Calaguala. 8vo. Pavia, 1791.

The plant which is the ſubject of this Eſſay, was firſt mentioned in the London Medical Journal, Vol. V. p. 423. Its botanical hiſtory has not yet been ſatisfactorily aſcertained, but it is ſaid to be a ſpecies of fern growing in Quito. For ſeveral years it has been much employed in different parts of Italy, particularly in the Duchy of Mantua, in inflammatory complaints, in catarrhs, phthiſis, and dropſy. The Academy of Sciences at Mantua, were therefore induced, in 1790, to make it the ſubject of a prize queſtion, the objects of which were to determine the chemical and medicinal properties ties of this plant. Signor Carminati, Professor of Physic at Pavia, made some inquiries relative to this subject, the results of which he has given to the public in this Essay, but without having aspired to the prize. He gives a chemical analysis of the root in question, and describes its sensible qualities, together with its effects in different diseases.

159. Sulla polmonare Tisichezza; Dissertazione epistolare indirizzata al Nobile Signore *Benigno Cannella* celebratissimo Professore di Medicina e Chirurgia nella Citta di Riva, da *Luigi Francesco Castellani*, Medico primario dell' Archiospedale di Mantova, e Prof. di Medic. Prattica. 8vo. Mantua, 1791.

160. Lettere Fisico-Mediche del Dottor *Francesco Vacca Berlinghieri*, Professore nell' Università di Pisa. 8vo. Pisa, 1791.

161. La Esplorazione proposta come fondamento dell' Arte Ostetricia da D. *Vincenzo Malacarne*, Saluzzese P. P. d'Istituzioni Chirurgiche e d'Ostetricia nell' Università di Pavia. 8vo. Milan, 1791.

162. Paralleli tra la Pellagra ed alcune Malattie, che piu le rassomigliano, del Dottor *Francesco Fanzago*. 8vo. Padua, 1792.

### INDEX.

### A.

ABSORBENT Syſtem, Work relative to, 209

Acid, vegetable, Treatiſe on the Effects of, 217

Ackerman, J. F. Ueber die Cretinen, —— 215

——, J. C. K. Medicin. Skizzen, —— 216

Æther, Vitriolic, its Effects in Spaſm of the Stomach 68

—— in Intermittents, 71, 75, 77

—— in Epilepſy, —— 78

Almonds, Effects of, —— —— 60, 63

Amenorrhœa, Diſſertation on, —— 202

Amputation, immediate, in compound Fractures, Arguments againſt, —— —— 52

Amſterdam, Eſſays by the Agricultural Society at, 213

Anatomy, morbid, Treatiſe on, —— 196

——, Treatiſe on, —— 220

Aneuriſm, popliteal, Caſes of, —— 3, 11

Anguſtura Bark, Works relative to it, — 194, 210

Animation ſuſpended, Works relative to, 199, 201, 203

Anthelmintics, Works relative to, —— 204, 206

Apoplexy, Caſe of, in a pregnant Woman, — 96

——, Diſſertations on, —— 201, 203

Arnold, J. D. de Suppuratione, —— 210

Aſcites, Diſſertations on, —— 201, 203

Aſh, Joann. Oratio Harveiana, —— 200

Aſtragalus, Caſe of Fracture of, —— 44

—— Rotation of, —— 54

Auſmkolk, F. W. de Cortice Caribæo, — 203

### B.

Baillie, Dr. M. morbid Anatomy, —— 196

Balme, M. Mem. de Medecine pratique, — 212

Bandages, ſurgical, Treatiſe on, —— 215

Bark, Obſ. on the Uſe of in Fevers, —— 234

——, Caribæan, Diſſertation on, —— 203

——, red, Obſervations relative to it, —— 156

——. *See* Anguſtura.

Barry, Joannes, de Aſcite, —— 201

Barytes, muriated, Works relative to, — 209, 220

Bath

Bath Waters, Treatiſe on, —— 190

Baths, Treatiſe on, —— —— 220

Beddoes, Dr. Tho. on the poiſonous Effects of Digitalis, 17

———— on Calculus, &c. —— 194

———— on pulmonary Conſumption, 195

Behrends, J. B. J. Diſſert. de Corde, —— 205

Bengal, Obſ. made in a Voyage to, —— 20

Berlinghieri, F. V. Lettere Fiſico.Med. —— 222

Bibliography, Medical, Works relative to, 202, 203

Binham, Geo. J. de Gonorrhœa, —— 201

Bitter Aſh. *See* Quaſſia Polygama.

Bitter, vegetable, remarkable Effects of, —— 60

————, ſuppoſed to poſſeſs a narcotic Quality, 67

Bitter Wood. *See* Quaſſia Polygama.

Bluhm, H. on the Diſeaſes of Reval —— 214

Blumenbach, J. F. his Obſ. on the pigmentum of the Eye referred to, —— —— 160

———— Eſſay on Generation, — 192

Blumenberg, C. F. de Cort. Anguſturæ, —— 210

Bolton, Joannes, de Apoplexia, —— 201

Bonhard, G. Chr. de uſu Lienis, —— 204

Botany, Works relative to, — 193, 199, 200, 206

Böttcher, J. F. vermiſchte Schriften, — 218

Brande, A. E. on the Anguſtura Bark, —— 194

Braſchiæ (plantæ nov. gener.) Deſcript. — 206

Briſtol Waters, Work relative to, —— 194

Broſig, P. S. de Febre nervoſa, —— 210

Bucholz, W. H. S. on Medical Juriſprudence, 215

Buettner, D. F. Semeiologiæ Rudimenta, —— 206

Bullen, Gulielmus, de Scrophula, —— 201

Bulliard, M. ſur les Champignons, —— 212

Burrowes, Dr. Geo. Account of a fiſtulous Opening in the Stomach, 185

Buxton Waters, Work relative to, —— 199

C.

Calaguala, Eſſay on the Root ſo called, —— 221

Calculus, Urinary, Works relative to, 194, 196

Carlſbad, Work on the Mineral Waters of, — 215

Carminati, Baſſ. Hygiene Therapeutice, —— 206

———— on the Root called Calaguala, 221

Caſtellani, L. F. ſulla Tiſichezza, —— 222

Cataracts, Treatiſe on, —— 192

Caw, Jac. Drew M', de Rheumatifmo, — 202
Chemnitz, J. A. de Morbis Venereis, — 210
Chin Cough, Work relative to, — 218
Cinchona Brachycarpa, botanical Defcription of, 152
———, its medicinal Properties, 155
Clarke, Dr. J. Effays on Pregnancy and Labour, 197
Cold Bathing, Obf. on, — 192
College, Veterinary, Work relative to it, — 194
Concuffion of the Brain, Obf. on — 180
———, three Claffes of, diftinguifhed, 182
Confumption, Works relative to it, 194, 195, 222
Covillard, his Account of a fiftulous Opening of the Stomach, — — 189
Cows, Effay on the Parturition of, — 215
Cretins, Work relative to, — 214
Creve, C. C. de Fracturis Offium Pelvis, — 205
Crichton, Dr. Alex. his Tranflation of an Effay on Generation, — — 192
Currie, Dr. James, on the Effects of a Shipwreck on the Mariners, — — 103
Curry, Dr. James, on fufpended Animation, 159

D.

Danz, F. G. Gefchichte des Keichhuftens, — 218
Datura Stramonium, Effects of the Seeds of, — 78
Davidfon, Jac. de Vita Submerforum refufcit. 201
———, W. on the Effects of Vitriol. Æther, 68
Deafnefs, Differtation on, — 204
Deafe, Mr. his Work on Wounds of the Head recommended, — — 178
Denman, Dr. Jof. on Buxton Waters, — 200
Diamond Harbour, its unhealthy Situation, — 30
Diet, antifcorbutic, Obf. relative to it, — 35
Digitalis purpurea, Account of the poifonous Effects of, 18
———, Work relative to its Effects in Dropfy, 190
Dovering, S. J. L. de Vomitu, — 209
Droop, J. D. de Viribus Medicamentorum, — 203
Dropfy of the Brain, Treatife on, — 190
Duifburg, C. G. L. de Phyfiologia et Pathol. Uteri, 207
Dyfentery, Differtations on, — 201, 202
Dyfpepfia, Differtations on, — 201, 202

E.

Earle, James, on the Hydrocele, —— 196

————, on Lithotomy —— *ibid.*

East Indies, Obs. made in a Voyage to, —— 21

Eberhard, J. G. on the Parturition of Cows, 213

Eckner, G. C. Hist. of a bilious Fever, —— 214

Edinburgh, Cat. of the Library of the Coll. of Phys. at, 202

————, Guide for medical Students at, — 192

Eggert, J. D. de Geoffræa Surinam. —— 206

Electricity, animal, Works relative to, 193, 194

Epilepsy relieved by Vitriolic Æther, —— 98

Essera, Distinction between it and Urticaria, — 65

Esten, Joannes, de Febre flava, —— 201

Eyerel, Jos. his Edition of Prof. Stoll's Works, 205

Eyes, singular Construction of, in a Girl, — 158

F.

Fabricius, L. C. de Signis Morb. ex Oculorum Habitu, 209

Fachinger, Description of the Waters of, —— 219

Falconer, Dr. W. Diss. on the Bath Waters, — 190

Fanzago, Fr. sopra la Pellagra, —— 222

Farriery, Lectures on the Elements of, —— 198

Fever, bilious, epidemic, Hist. of, — 213

——, remittent, Cases of, —— 38, 40

——, putrid, Works relative to, —— 196

——, Yellow, Dissertation on, —— 201

Finighin, an ancient Irish Surgeon, Anecdote of, 162

Foot, Case of Distortion of, —— 54

Foot, Jesse, on the Prevention of Hydrophobia, 200

Forceps, Obstetric, Work relative to, —— 197

Ford, Edw. Account of a Child without Organs of Generation —— —— 92

———— on the Disease of the Hip Joint, 200

Forster, Thompson, Cases of Popliteal Aneurism, 3, 11

Fowler, Richard, on animal Electricity, —— 194

Frize, F. H. Annalen der Klinisch. Inst. zu Berlin, 219

———— ueber die Vener. Krankheit, — 219

G.

Gagel, J. E. de Suicidio, —— 204
Ganne, Ambr. l'Homme Physique et Moral, 211
Gautier, Lud. de Irritabilitate, —— 209

Gardinius, Jof. de Electrico Igne, — 209
Generation, Effay on, — 192
Gefenius, W. Catal. of fimple Remedies, — 217
Girtanner, C. Neue Chem. Nomenklature, — 219
Gmelin, J. F. Materia Medica, — 217
Goethe, J. W. von, on the Metamorph. of Plants, 215
Gonorrhœa, Works relative to, — 201, 211
Goodman, Geo. de Scorbuto, — 201
Gordon, Alex. Indigenous Botany, — 199
Graves, Dr. Robert, on the Nottington Water, 191
Guy, William, Cafe of Diftortion of the Foot, 54

H.

Halloran, Sylv. O', on the Ufe of the Trephine, 161
Haygarth, Dr. John, Plan to exterminate the Small Pox from Great Britain, — — 191
Head, Injuries of, that require the Trephine, Obf. on, 161
Heat, animal, Experiments to afcertain the Effects of Immerfion in Water on, — 111
——, Obf. relative to, — 125
Hebenftreit, E. B. G. ueber der Med. Polizey, 219
Hip-Joint, Obf. on the Difeafe of, — 200
Holme, Edvard. de Vaf. Abforbent. — 209
Hofers, F. J. on Surgical Bandages, — 215
Hofpitals, Work on the Advantages of, — 214
Hufeland, C. W. ueber der Sauren Schwererde, 220
Hunter, John, his Obf. on the Colour of the Pigmentum of the Eye quoted, — — 151
Hydrocele, Work relative to, — 196
Hydrocephalus, Treatifes on, — 190, 203
Hydrophobia, Cafe of, — 87
——, Plan for preventing it, — 200

I.

James, Samuel, Obf. on the Willow Bark, — 191
Jaundice, Differtation on, — 202
Immerfion in hot and cold Water, Effects of, on the living Body, — — 110
Intermittents, may be cured by exciting Commotion in the Body, — — 70, 74
——, cured by Vitriolie Æther, 71, 75
John, J. G. de Medic. Syft. lymph. fubmovent. 204
Johnfon, J. (a fictitious Name) Guide for medical Students at Edinburgh, — — 192

Johnfon, James, on the Effects of the Seed of the Stramonium, ——— ——— 78
———, Dr. R. W. Cautions to Families, — 198
Itch, Work relative to, ——— 218
Jurifprudence, medical, Works relative to, 204, 214, 215

K.

Kampf, Joh. Enchiridium medicum, —— 207
Karch, Gottl. de ufu Thymi, —— 204
Khittel, Euft. a, de Scarlatina, —— 210
Kilburn Wells, Analyfis of the Water of, —— 100
Klaproth, M. H. on the Carlfbad Waters, —— 215
Kleefeld, J. G. Differt. Inaug. de Pathemat. 204
Kommer, J. P. de Hydrometra, ——— 205
Kritter, J. F. de Auditu difficili, ——— 204

L.

Ledger, Gul. de Dyfpepfia, ——— 201
Lewis, Gul. B. de Dyfenteria, ——— 201
Lindfay, John, Account of the Quaffia Polygama, 140
——————— Cinchona Brachycarpa, 152
Liver, State of, its Influence in Difeafes of hot Climates, 33
———, Treatife on its Economy and Difeafes, 194
Lobes, E. F. G. von, on the Itch, ——— 218
Lobftein, Dr. on the Effects of the Stramonium, 82
Lodemann, J. G, de Hydrocephalo, —— 203
Loefeke, J. L. L. Materia Medica, ——. 216
Lund, Mr. N. Tönder, of the true Quaffia Amara, 145

M.

Madrafs, Obf. made in a Voyage to, ——— 21
Malacarne, Vinc. fopra la Efplorazione, ——— 222
Mann, Abbé, fur les grandes Gelées, — 213
Marcard, H. M. ueber die Bäder, ——— . 220
Marcus, A. F. on the Advantages of Hofpitals 214
Marfchal, F. L. on Caftration, ——— 218
Martelli, Nic. Defcr. Brafchiæ Plantæ nov. Generis, 206
Martyn, Tho. Language of Botany, ——— 199
Materia Medica, Works relative to, 192, 193, 203, 206, 210, 216, 221
Meckel, Prof. neue Archiv der Prakt. Arzneyk. 216
Melitch, J. on the retroverted Uterus, — 215
Mercury, combined with Bark, in Fevers, Ufe of, 33

Metzger, J. D. Exercitat. Acad. —— 208

Michaelis, P. on the Decuſſation of the Optic Nerves, 216

Midwifery, Works relative to, 197, 213, 215, 220, 222

Miller, Joſephus, de Aſcite, —— 203

Milne, Dr. Colin, Indigenous Botany, —— 199

Mineral Waters, Works relative to, 100, 190, 191, 215, 219

Moore, James, Eſſay on the Materia Medica, 192

Murray, Jo. And. Enum. Lib. præcip. Argum. med. 203

—————— Apparatus Medicaminum, 203

N.

Natural Hiſtory, Inſtitutes of, —— 191

Necker, N. J. de, Phytozoologie Philoſ. —— 211

Nerves, Optic, Work on the Decuſſation of, 216

Nettle Raſh, Caſes of, —— 57

Neurology, Work relative to, —— 206

Nitre, in Fumigation, good Effects of, —— 24

Nott, Dr. John, on the Briſtol Waters, —— 194

Nottington Water, Work relative to it, — 191

O.

Olivari, Nicol. Piano della Scuola Clinica, — 221

Opium, good Effects of, in a Perſon poiſoned by Digitalis, 18

P.

Patience, in Attendance on the Sick, Eſſay on, 218

Pavia, Pharmacopœia of the Hoſpital at, — 205

Peacock, H. B. Treatiſe on Cataracts, —— 192

Pellagra, Work relative to it, —— 222

Pelvis, Diſſert. on Fractures of the Bones of, 205

Perceval, Charles, Account of a Girl whoſe Eyes are conſtructed in an extraordinary Manner, — 158

Petit, M. Ant. Projet de Reforme de la Med. 210

Pharmacopœia of London, poſologic Companion to, 198

—————— of Bremen, —— 207

—————— of Lippe, —— 208

—————— of the Hoſpital at Pavia, —— 205

Phthiſis, Diſſert. on, — — 222

Phyſic, Materials for the Hiſtory of, — 216

Phyſick, Phil. Syng, de Apoplexia, —— 203

Pigmentum of the Eye, Obſ. relative to it, — 151

Plants, Metamorphoſes of, Work relative to, 215

Plinta, J. M. Hiſt. Epidem. varioloſ. — 208

Police,

Police, medical, Treatife on, — 215, 219
Popliteal Aneurifm, Cafes of, — 3, 11
Preu, P. S. C. Epiftola gratulatoria, — 207
Prickly Heat, Obf. relative to it, — 67

Q.

Quaffia Amara, fpoken of as a very rare Plant, 145
———, a fpurious Sort of, has been imported into Europe for the true, — *ibid.*
——— Polygama, Account of, — 140
———, defcribed under the Name of Picrania Amara by Dr. Wright, — — 142
———, the Quaffia Excelfa of Dr. Swartz, 142, 148
Quin, Dr. C. W. on Dropfy of the Brain, — 190

R.

Rawlins, R. on the Obftetric Forceps, — 197
Reil, J. C. Memorab. med. practica, — 209
Relhan, R. Floræ Cantabr. Suppl. — 200
Rels, H. W. Onomatologia Chymica, — 219
Reval, Work on the Difeafes of, — 213
Rheumatifm, acute, Differtation on, — 202
Rohr, Mr. Von, his Account of the true Quaffia Amara, 145
Rowley, Dr. W. on Scarlatina Anginofa, — 196
Rumfey, James, Cafe of a Diflocation of the Tibia and Fibula, — — 44
Rufh, Dr. of the Effects of Stramonium, — 80
Rüft, C. J. F. de Infarctus Differentiis, — 204
Ryan, Dr. M. on the Afthma, — 195

S.

Sainbel, C. Vial de, Elements of Farriery, 194
Salix Latifolia, Bark of, Work relative to it, 194
Saunders, Dr. W. Treatife on the Liver, — 190
Scarlatina, Work relative to, — 213
Scherf, J. C. M. Difpenfat. Lippiacum, — 208
Schlegel, J. C. T. Thefaur. Semeiol. Pathol. 207
Schmeiffer, J. G. Defcription of Kilburn Wells, 100
Schmidt, J. de, de Baryte Muriato, — 209
Schwartze, A. J. de Cortice Geoffrææ Surinam. 204
Scrophula, Differtation on, — 201

Scurvy,

Scurvy, Sea, Obf. and Works relative to it, 36, 123, 194, 201, 202, 208
——————, Dyfpnœa an alarming Symptom in, how relieved, —— —— 36
Seifert, P. D. B. de Annis Climactericis, — 205
Semeiology, Works relative to, — 206, 207, 209
Shipwreck, remarkable Effects of, on the Mariners, 103
Simon, Salom. de Profopalgia, —— 210
Simmons, Richard, Cafe of Hydrophobia, — 87
Simpfon, William, Obf. on Cold Bathing, — 192
Small Pox, Works relative to, 191, 195, 198, 208, 212
Société d'Hiftoire Natur. de Paris, Actes de la, 212
Soemmering, S. T. Works by, —— 216, 220
Spleen, Differt. on the Ufe of, —— 204
Stennet, Joannes, de Dyfenteria, —— 203
Stoll, Maxim. de Morbis chronicis, —— 205
—————— Aphorifmi de Febribus, —— *ibid.*
Stomach, fiftulous Opening in, Inftances of, 185, 189
————, Work relative to its Connection with Life, 198
Störck, Anton. B. de, Præcepta medico-practica, 206
Stramonium. *See* Datura.
Stupor, immediately following a Fall, defcribed as a certain Sign of Concuffion of the Brain, —— 181
Swartz, Dr. Olof, his Defcript. of the Quaffia Excelfa, 148

T.

Temple, Dr. R. Practice of Phyfic, —— 195
Teytaud, F. de la Gonorrhée, —— 211
Thelwall, John, on animal Vitality, —— 197
Thiery, M. Obf. de Phyfique et de Med. — 212
Thilenius, M. G. Befch. des Fachinger Mineral-Waffer, 219
Thorn Apple. *See* Datura.
Thymus, Differtation on its Ufe, —— 204
Trephine, Obf. on the Ufe of, —— 161
Trotter, Dr. Tho. Obf. on the Scurvy, —— 193

U.

Valli, Dr. Eufebius, on animal Electricity, — 193
Vavafour, Gulielm. de Ictero, —— 203
Venereal Difeafe, Works relative to, 210, 211, 212, 219
Verginita, dei Segna della, —— 220
Vitality, animal, Differt. on, —— 197
Urticaria, Cafes of, — —— 57

Urticaria, Obſ. on, —— — 64

Uterus, Diſſertation on the Phyſiology and Pathology of, 207

——, retroverted, Treatiſe on, —— 215

W.

Waldkirch, J. U. de Aſphyxia Neonatorum, 204

Walker, Dr. Inſtitutes of Natural Hiſtory, — 191

Watſon, John, his Obſ. on Diſeaſes in a Voyage to the Eaſt Indies, —— —— 21

Webſter, Dr. C. on the Connection of the Stomach with Life, —— —— 198

Weems, Joannes, de Amenorrhœa, —— 202

Wezeler, F. G. on Med. Juriſprudence, —— 214

Williams, P. Caſe of Apoplexy in a pregnant Woman, 96

Willow, Bark of a particular Species of, Work relative to, 192

Wilſon, Alex. Phil. de Dyſpepſia, —— 203

——, Samuel, de Variolis, —— 203

Winterbottom, Dr. T. M. Obſ. on Urticaria, 57

Wittwer, P. L, ueber die Geſchichte der Arzneyk. 216

Woenſel, Dr. F. Van, on the ſmall Pox, — 195

Woodville, Dr. W. Medical Botany —— 193

END OF THE FIFTH VOLUME.

